# Calculated parenteral initial treatment of bacterial infections: Economic aspects of antibiotic treatment

**DOI:** 10.3205/id000047

**Published:** 2020-03-26

**Authors:** Michael Wilke, Claudia Hübner, Wolfgang Kämmerer

**Affiliations:** 1 inspiring-health Dr. Wilke GmbH, Munich, Germany; 2 Lehrstuhl für Allgemeine Betriebswirtschaftslehre und Gesundheitsmanagement, Universität Greifswald, Germany; 3 Klinische Pharmazie, Apotheke des Universitätsklinikums Augsburg, Germany

## Abstract

This is the seventeenth chapter of the guideline “Calculated initial parenteral treatment of bacterial infections in adults – update 2018” in the 2^nd^ updated version. The German guideline by the Paul-Ehrlich-Gesellschaft für Chemotherapie e.V. (PEG) has been translated to address an international audience.

This chapter analyses economic aspects of antiinfective therapy. Any treatment decision is also a cost decision. In this chapter the authors particularly analyse whether or not there is evidence that certain clinically effective strategies as Antimicrobial Stewardship programs (AMS), guideline adherent initial therapy, early diagnostics, De-escalation, sequence therapy or therapeutic drug monitoring also have benficial economic effects. These can be direct savings or shortening of length of stay to free resources.

## Zusammenfassung

Dies ist das siebzehnte Kapitel der von der Paul-Ehrlich-Gesellschaft für Chemotherapie e.V. (PEG) herausgegebenen S2k Leitlinie „Kalkulierte parenterale Initialtherapie bakterieller Erkrankungen bei Erwachsenen – Update 2018“ in der 2. aktualisierten Fassung.

Dieses Kapitel setzt sich mit den ökonomischen Effekten der antiinfektiven Therapie auseinander. Jede Behandlungsentscheidung ist auch eine kostenrelevante Entscheidung. Die Autoren beleuchten insbesondere die Frage, ob es Evidenz dafür gibt, das klinische wirksame Strategien auch ökonomisch günstig sind. Unter anderem werden Antibiotic Stewardship Programme (ABS), Leitlinienadhärenz in der Initialtherapie, De-Eskalation, Sequenztherapie sowie das therapeutische Drug Monitoring. Ökonomisch günstig sind sowohl direkte Kosteneinsparungen als auch die Verkürzung der Verweildauer um Ressourcen schneller wieder bereit zu stellen.

## Introduction

Calculated parenteral initial treatment of bacterial diseases in adults aims at choosing the right antibiotic at the earliest possible moment in order to maximize the chances of curing the infection. In addition, the recommendations for calculated treatment should also contribute to minimizing risk of developing resistance. In the following, the economic aspects of antibiotic treatment will be analyzed and strategies presented that are favorable from an economic point of view. Practically all studies and publications on the economic evaluation of certain antibiotic treatment strategies show that clinical (time to cure, survival, proportion of superinfections) and economic benefits go hand in hand. Thus, none of the economically favorable treatment strategies presented here have a negative impact on the clinical outcome. 

In most European countries, including the German-speaking countries, remuneration systems based on the so-called “diagnosis-related groups (DRG)” are in use in hospitals. These systems have in common that they reimburse for a hospital stay on the basis of the principal diagnosis, interventions performed (surgeries and other procedures) and any secondary diagnoses (for example nosocomial infections). In these compensation systems in particular, all diagnostic and therapeutic strategies that lead to a longer hospital stay from the outset are economically unfavorable, since reimbursement usually takes the form of a per-case lump sum based on the average cost of a patient, which in turn is strongly influenced by the average length of stay. If the in-patient stay extends beyond the average length of stay, treatment usually costs more than the reimbursement for the case. In classical pharmacoeconomics, considerations of drug costs are often in the foreground. Since these costs generally only account for about 4% of the costs in a hospital (intensive care wards 10%), they are well below the costs associated with longer stays. Nevertheless, strategies should also be considered that lead to a reduction in the cost of medicines through targeted intervention. Finally, the authors have included a simple guide in this text that readers can use to perform their own analyzes.

Pharmacoeconomic parameters such as cost-effectiveness or costs per quality-adjusted year of age (Cost/QALY) are not considered, since these considerations do not play a major role in German-speaking countries and are only used in some English-speaking countries to decide whether or not certain medicines should be reimbursed.

The aim of this text is to give readers a quick overview of economically advisable strategies in the form of a table, which in addition to the strategies also contains a level of recommendation in order to decide which of them should be used systematically (see Table 1 (Tab. 1)).

## Diagnostic and therapeutic strategies in detail

### Adequate initial therapy

The selection of the antibiotic at the beginning of treatment – especially in critically ill patients – determines the clinical and economic outcome to a high degree. Inadequate treatment is associated with significantly higher mortality [1], [2], [3], [4], [5] and usually higher costs [1], [6], [7], [8], [9]. However, the term “inadequate treatment” is rather vague. In the following we present aspects, including examples from the literature, which individually or in combination lead to inadequate initial therapy.

### Compliance with guidelines

Guidelines and recommendations combine diagnostic and therapeutic strategies designed to ensure that the most common pathogens in certain infections – factoring in the current resistance situation – are included in initial treatment. Thus, adherence to guidelines, including local recommendations based on national and international guidelines, is an important driver of clinical and economic outcomes of treatment. There are examples in the literature of prospective randomized studies [10], [11], [12], case-control studies and so-called “interrupted time series” analyzes – more commonly known as “before and after” [13], [14], [15], [16]. After reviewing the literature and assessing the evidence, the authors strongly recommend (A) adherence to guidelines.

### Consideration of the local resistance situation and patient-specific risk regarding the presence of resistant pathogens

Inadequate antibiotic treatment is associated with increased mortality and prolonged hospital stays [2], [9], [17]. Therefore, it is relevant not only from a clinical but also from an economic point of view that adequate antibiotic treatment takes place as soon as possible, ideally starting with calculated initial treatment.

In addition, many studies comparing adequate and inadequate treatment have found in particular that patients who were proven to have infections with multidrug-resistant pathogens often did not received adequate initial treatment [15], [18], [19], [20], [21], [22], [23], [24], [25].

The resistance rate, i.e. the proportion of strains of a bacterial species that is resistant to one or more antimicrobial substances, has been identified as a factor that influences the cost-effectiveness of antibiotics [26]. The effects were investigated in various decision analysis studies using the example of community-acquired pneumonia (CAP) [27], [28], [29]. Sensitivity analyzes showed that taking the resistance rates of *Streptococcus* *pneumoniae* and *Haemophilus* *influenzae* into account in the selection of active agents resulted in a reduction of the failure rate of first-line treatment (thus removing the need for second-line therapy), hospital admissions and mortality.

In the treatment of life-threatening bacterial infections in which calculated antibiotic treatment is used initially, knowledge of the local, often even ward-specific pathogen spectrum and associated resistance situation is crucial. Therefore, it is essential that the resistance statistics are continuously compiled, evaluated and communicated to the clinicians by the microbiologists (or hospital hygienists). Microbiological diagnostics plays a special role in this. It has two important functions to fulfill:

- Modification of initially calculated antibiotic treatment through microbiological findings and
- Providing data for determining the local pathogen and resistance spectrum against which future calculated antibiotic strategies will be targeted.

Especially regarding the first point it is important to obtain microbiological results as soon as possible. Here the use of fast and expensive diagnostic procedures can indeed be justified economically [30], [31]. The aim is to initiate early escalation or de-escalation of calculated initial treatment by quickly determining the resistance status and thus to reduce the duration of possibly inadequate treatment with its associated negative consequences. Cost efficiency has been demonstrated in economic model calculations, for example for PCR-controlled calculated antibiotic treatment [32], [33].

In addition, it is important to consider patient-specific risk factors that indicate infection with a multidrug-resistant pathogen when selecting treatment. These primarily include previous treatment with antibiotics, colonization or infection with an MRE or pathogen with special resistance in the medical history, a hospital-acquired infection or a previous hospital stay, chronic immunosuppression (cancer, COPD, diabetes, MTX therapy with PCP, etc.) as well as stay on an intensive care ward (possibly with ventilation) and acute or chronic renal failure, to name only the most important [34], [35], [36], [37], [38], [39]. The evaluation/weighting of such risk factors is recommended, amongst others, in the selection of appropriate antibiotics for calculated initial treatment of pneumonia [40], [41], [42].

Failure to take account of the risks leads to poorer clinical outcomes and higher treatment costs. In these patients, the choice of an antibiotic which acts against multidrug-resistant pathogens in initial therapy may be the better choice clinically and economically. As soon as the pathogen is known, treatment should be adapted accordingly i.e. de-escalated. 

The benefit of early consideration of multidrug-resistant pathogens in high-risk patients has so far only been shown in retrospective case-control studies but the authors nevertheless strongly recommend (A) this strategy.

### Rapid diagnostics with modern methods

Precisely because misjudging the risk of a certain pathogen being present often leads to inadequate initial therapy and because pathogen identification by means of culture in clinical practice takes 48 hours or longer, the question arises as to whether newer diagnostic methods such as real-time PCR, MALDI-TOF or the PCR-based electron spray mass spectroscopy (PCR/ESI-MS) [43] can contribute to adequate initial therapy and a reduction in costs. Since these procedures are very expensive compared to conventional diagnostics, the question arises of when they are useful. Various authors have carried out investigations and selected different scientific approaches (expert assessment on the basis of test results [44], [45], modeling [33], before/after [30], [31], [46]). One work showed that rapid testing led to a reduction in the use of vancomycin and shortened the duration of hospital stays [47]. After assessing the evidence for the present work, which deals explicitly with the economic effects of rapid diagnostics, the authors give a medium recommendation for this strategy (B).

### Antibiotic stewardship programme (ABS)

Many measures to optimize antibiotic treatment can be subsumed under the term ABS. Here it was analyzed if there is evidence that extensive programs with measures such as

- creation of in-house recommendations,
- regular prescription analysis with ward rounds and continuous feedback,
- advice from ABS experts (such as infectiologists or clinical pharmacists), and
- restriction of certain antibiotic classes

are clinically and economically sensible. A number of international authors emphasize this clearly [48], [49], [50]. In 2013 a Cochrane Review [51] and an S3 guideline on this topic [52] appeared. Overall, according to the authors, the evidence for the introduction of ABS programs and their clinical and economic benefits is very good and they are strongly recommended (A).

### Sequential therapy

Parenteral-oral follow-up treatment (sequential therapy) gives the option to continue treatment initiated parenterally in hospital with oral (out-patient) administration. As a result, the duration of intravenous treatment is reduced without having a negative impact on the success of the treatment [53]. In addition to reducing the risk of infusion-related infection and mobilizing the patient more quickly, there are a number of economic advantages that speak in favor of sequential therapy.

An early move to oral drug forms leads to a significant reduction in hospital stays, which can play a significant role in DRG flat-rate hospital remuneration systems. In a Europe-wide retrospective analysis of the treatment of MRSA-associated skin and soft tissue infections the team led by Nathwani and Eckmann found, for example, that the introduction of sequential therapy shortened hospital stays by 6.2 days on average with resultant potential savings of €2,000 per patient [54]. Similar results were reported by Gray et al. with her study in 5 hospitals in the United Kingdom, where she found savings of £363 per patient [55].

Other reasons for the economic superiority of sequential therapy over continuous parenteral therapy can be lower antibiotic costs and lower personnel costs for the preparation and administration of the parenteral antibiotics. The effects are not only evident in the clinical area but also in pre- and post-inpatient care.

Although there are mainly retrospective studies available on sequential therapy and its economic advantages, from an economic point of view the authors strongly recommend it (A).

### De-escalation

In addition to sequential therapy, de-escalation can also contribute to optimizing the clinical economic balance. The aim is to replace calculated initial broad-spectrum antibiotic treatment with a more targeted one, i.e. to replace the initial substance with a similarly effective substance that however has a narrower spectrum. Prerequisites for this are:

- presence of specific and plausible microbiological findings
- clinical improvement (patient responded well to initial treatment)

By reducing the treatment spectrum and thus the antibiotic load, the development of resistances should be influenced favorably by minimizing the selection pressure. Patient safety is improved through fewer adverse drug reactions and superinfections [52]. From an economic point of view this will result in (sometimes significant) savings in drug expenditure, not least by reducing the duration of treatment [56].

As with sequential therapy, the publications on the economic effects of de-escalation are predominantly either retrospective analyzes or secondary evaluations of clinical studies. Nevertheless, once again the authors express a strong recommendation (A).

### Therapeutic drug monitoring (TDM)

It is important to determine drug levels, particularly in the case of antibiotics with a narrow therapeutic range, such as vancomycin but also in the case of prolonged treatment with beta-lactam antibiotics. For TDM in vancomycin it has been shown repeatedly that using TDM significantly reduces nephrotoxic complications and thus, despite the costs, leads to considerable savings through avoiding complications [57], [58].

An analysis of 200 intensive care patients with severe infections investigated various therapeutic strategies with piperacillin/tazobactam. With an average total cost of €90.64 for a 7-day treatment with piperacillin/tazobactam, in spite of the additional costs of therapeutic drug monitoring (TDM, €26.68) continuous administration of an individual dose was below the cost of intermittent bolus administration in line with the package insert recommendations of 3x 4.5 g (for complicated urinary tract infection, intra-abdominal infections, skin and soft tissue infections, €112.11) or 4x 4.5 g (for severe pneumonia, neutropenic adults with fever, in cases of suspected bacterial infection, €148.49). Reduced drug costs contributed to this result – €36.75 [3x 4.5 g]/€49.00 [4x 4.5 g] bolus application versus €24.50 [8 g (2–16 g), median (min, max)] continuous application with TDM – about 30–50%. Plus on the other hand the lower process costs (disposable items and working time for preparation and continuous application (€46.11/€61.48 bolus application versus €24.42 continuous application) [59].

Although one of the studies on vancomycin was a randomized clinical study, overall there are relatively few studies on the economic aspects of TDM, the authors recommendation is a B-grade.

### Importance of process costs

At the latest with the introduction of DRG, the analysis of their process costs and the resulting process optimization became imperative for hospitals. Here, it is important to consider the process of drug treatment from drug procurement through to administering it to a patient.

An important instrument for process optimization is the establishment of clinical treatment pathways and the creation of standard operating procedures (SOPs). With the help of these treatment paths/process descriptions it is possible to document and ensure cost and quality of treatment. Part of the treatment pathways are standards in drug therapy. Anti-infective agents are an important drug group because of their major importance in terms of cost and also their significance for the quality and success of treatment. These treatment standards are also an important part of ABS programs. 

An important criterion for the selection of appropriate anti-infective agents in the treatment pathways/processes will be the economic-pharmacoeconomic analysis of alternative treatments from the perspective of a hospital. In addition to the purchase prices of drugs, consumption of other resources must also be taken into account.

It should also be questioned to what extent the anti-infective agent used satisfies aspects of quality management, quality assurance, process management, patient orientation and employee orientation. The following parameters are therefore included in such an analysis:

- Personnel costs per application: under DRG conditions (increased output rates, reduced headcount), a reduction in the frequency of application should be considered positive. Also, the personnel costs incurred per application are an important criterion: they are given in the literature as being €2–4 or US$ per application [60], [61];
- the costs of the associated application aids such as syringes, cannulas, infusion sets, etc. In the literature, these costs are given depending on the type of application as being €1–4 [61];
- a lower error rate: Investigations and the resulting recommendations from English-speaking countries showed that the number of drug application errors decreases with the reduction of the frequency of application and the simplicity of preparation [62]. The required number of steps in preparation must also be taken into account. So, whenever possible, ready-made preparations should be used;
- the possible likelihood of confusion;
- the cost of required monitoring but also the reduction in the use of anti-infective agents through monitoring.

The aim of this process and process cost analysis is to improve quality while optimizing costs. Cost optimization in this sense means that with the help of the described analysis, an anti-infective agent is chosen from amongst equally good active ingredients that has the lowest resource consumption. Due to the small number of studies that deal explicitly with litigation costs and error costs in antibiotic treatment, the authors give a Grade B recommendation.

### Economic consequences with increasing frequency of resistance

From a clinical and ecological point of view, the risk of selection of antibiotic-resistant microorganisms should be minimized, as infections caused by multidrug-resistant or even pan-resistant bacteria are associated with a (considerably) increased mortality risk for patients. A number of publications on the health threat posed by antibiotic-resistant pathogens have also studied the associated costs. According to the State of the World’s Antibiotics report, the 23,000 patients who died as a result of an infection with resistant pathogens in the US led to health care costs of $20 billion and $35 billion in lost productivity [63]. The 2014 WHO Global Report on Antibiotic Resistance Surveillance includes, amongst other things, a systematic literature review of the cost of infections with resistant microorganisms. This very sophisticated report comes to the conclusion that the increase of resistant pathogens has led to increased costs but that no global extrapolation can be made on the base of existing data [64]. In particular, it is pointed out that the additional costs attributable to infections by resistant strains should be considered economically in comparison with infections by sensitive strains of a pathogen species in the same type of infection. For example there are several papers that have studied MRSA and MSSA infections economically. These gave attributable additional costs of €8,000 to €17,000 and US$13,900 respectively [65], [66], [67], [68], [69]. Based on these amounts, it is easy to understand how the figures in the extrapolation above were reached. They appear quite realistic. Another report concludes that the number of deaths from resistant pathogens will rise from 700,000 worldwide today to 10 million by 2050 if no further action is taken. In total, this would lead to a – global – total economic damage of US$100 billion (100 trillions US-notation in the original) by 2050 [70]. The authors acknowledge that first steps have been taken to tackle this global crisis. Intensified research, actions coordinated by WHO in 194 countries and advances in understanding the genetics of bacteria and, ultimately, the improvements in infection prevention in emerging economies are rays of hope.

In summary, it can be stated that resistance to antibiotics causes considerable direct financial and even greater economic damage. That is precisely why this topic should remain on the agenda in the future when discussing the economic aspects of antibiotic treatment.

### Further sources of information and their evaluation

In Medline, there are also increasing indications of health economic works. The journals, which mainly publish articles on health economic issues, include “Health Economics and Quality Management” published by the German Society for Health Economics as well as international English-language journals such as “Health Economics”, “European Journal of Health Economics” and “Value in Health”.

It is a well-known problem that not only approval-related treatment studies but also many pharmacoeconomic studies are carried out in cooperation with the pharmaceutical industry. Such studies tend to present positive results for usually high-priced drug innovations and are often used as marketing tools for external sales visits or at specialist congresses. Also the choice of a method of analysis is often result-oriented or extensive and non-transparent model calculations are used. For the non-economist it is difficult to recognize this publication bias and to classify the significance of such studies. One option is to focus on reports from Health Technology Assessment (HTA) agencies such as the National Institute for Health and Clinical Excellence (http://www.evidence.nhs.uk/), the Institute for Quality and Efficiency in Health (https://www.iqwig.de/) or the Canadian Agency for Drugs and Technologies in Health (http://www.cadth.ca/). In addition to a systematic presentation and qualitative assessment of the available evidence, these also include evaluations of the cost-effectiveness of pharmaceuticals and other medical technologies. These are based partly on existing studies and partly on their own economic studies. The increasing networking of international HTA agencies and a progressive standardization of assessment methods have an additional beneficial influence.

Studies and HTA reports are also presented in great detail in the database of the NHS Center for Review and Dissemination (http://www.crd.york.ac.uk/crdweb/). The NHS Economic Evaluation Database contains studies that can be found under Current Contents, Clinical Medicine, Medline, and CINAHL, as well as manual searches. Based on a scheme of approximately 30 criteria, the study objective, design of the clinical and economic part of the study as well as clinical and economic results are presented clearly and in detail. In addition, there is a brief evaluation of the study quality.

## Note

This is the seventeenth chapter of the guideline “Calculated initial parenteral treatment of bacterial infections in adults – update 2018” in the 2^nd^ updated version. The German guideline by the Paul-Ehrlich-Gesellschaft für Chemotherapie e.V. (PEG) has been translated to address an international audience.

## Competing interests

The authors declare that they have no competing interests.

## Figures and Tables

**Table 1:**
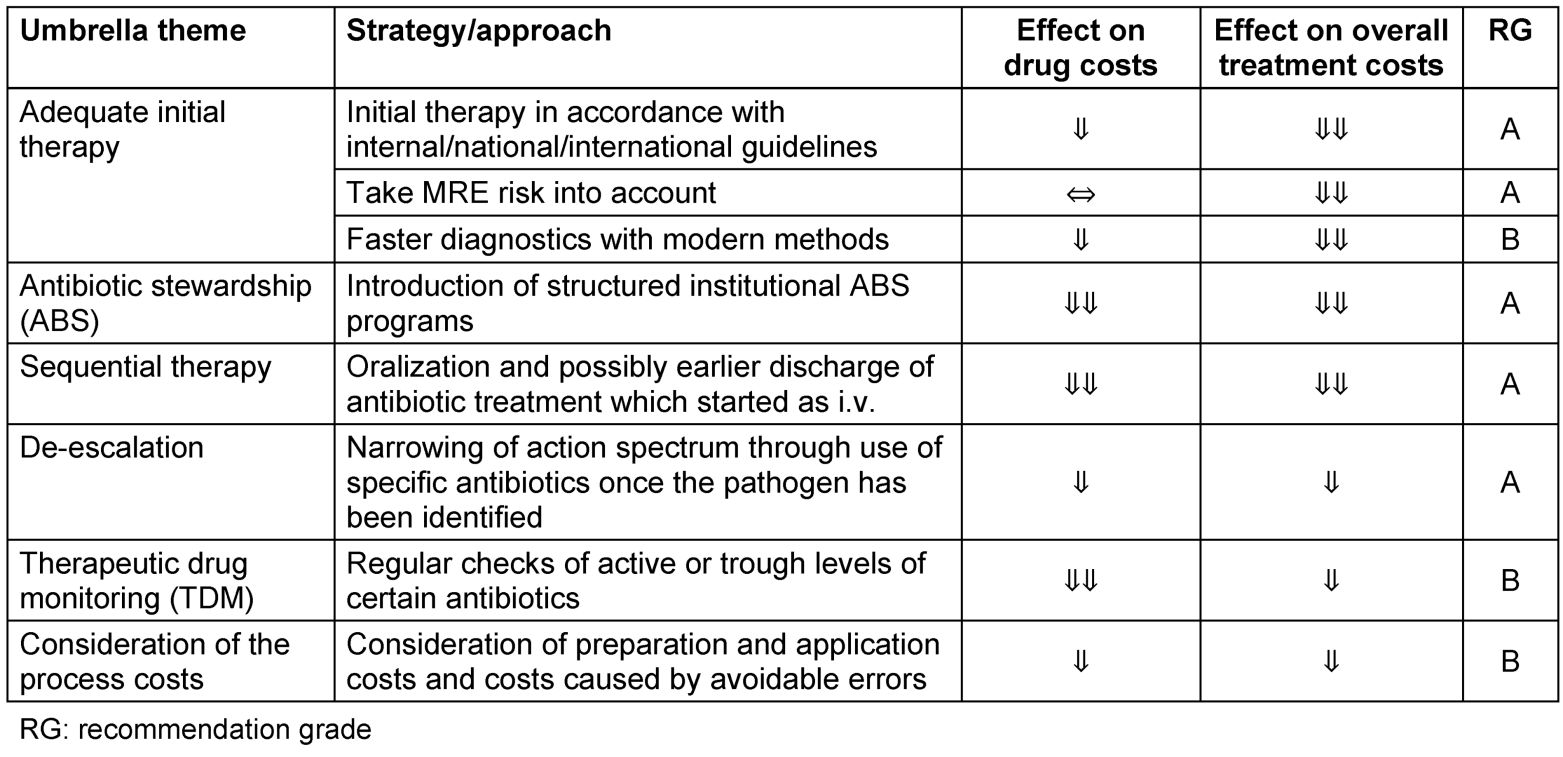
Overview of economically recommended strategies
